# Utilization, retention and bio-efficacy studies of PermaNet^® ^in selected villages in Buie and Fentalie districts of Ethiopia

**DOI:** 10.1186/1475-2875-8-114

**Published:** 2009-05-30

**Authors:** Messay Fettene, Meshesha Balkew, Ciara Gimblet

**Affiliations:** 1 Aklilu Lemma Institute of Pathobiology, Addis Ababa University, PO Box 1176, Addis Ababa, Ethiopia; 2 MIHRT program, Howard University, Washington DC, USA

## Abstract

**Background:**

Malaria remains a major public health problem in Ethiopia. Pyrethroid-treated mosquito nets are one of the major tools available for the prevention and control of malaria transmission. PermaNet^® ^is a long-lasting insecticide-treated net (LLIN) recommended by WHO for malaria control.

**Objective:**

The objective of the study was to assess utilization and retention of PermaNet^® ^nets distributed for malaria control in Buie and Fentalie districts and monitor the bio-efficacy of the nets using the WHO cone bioassay test procedures.

**Methods:**

A cross sectional study was carried out by interviewing household heads or their representative in Buie and Fentalie districts. The two districts were selected based on a priori knowledge of variations on ethnic background and housing construction. Clusters of houses were chosen within each of the study villages for selection of households. 20 households that had received one or more PermaNet^® ^nets were chosen randomly from the clusters in each village. A total of eight used PermaNet^® ^nets were collected for the bio-efficacy test. The bio-efficacy of PermaNet^® ^nets was monitored according to the standard WHO procedures using a susceptible colony of *Anopheles arabiensis *to deltamethrin.

**Results:**

A total of 119 household heads were interviewed during the study. The retention rate of nets that were distributed in 2005 and 2006 season was 72%. A total of 62.2% of the interviewees claimed children under five years of age slept under LLIN, while only 50.7% of the nets were observed to be hanged inside houses when used as a proxy indicator of usage of LLIN. For the bio-efficacy test the mean knock-down was 94% and 100%, while the mean mortality rate observed after 24 hr holding period was 72.2% and 67% for Buie and Fentalie districts respectively.

**Conclusion:**

The study revealed a moderately high retention of PermaNet^® ^in the study villages and effectiveness of the nets when tested according to the standard WHO procedure.

## Background

Malaria is a major public health problem in Ethiopia [1,2]. The country is most affected by malaria epidemics primarily due to its varying topographical and climatic features [3,4]. Malaria transmission in Ethiopia depends substantially on *Anopheles arabiensis *mosquitoes, a member of the *Anopheles gambiae *complex, in the intermediate highlands of Ethiopia [5].

Vector control remains the most effective measure to prevent malaria transmission and is one of the four basic technical elements of the Global Malaria Control strategy [6]. Insecticide-treated nets (ITNs) were reported to be effective in reducing all-cause of childhood mortality by 20%, and to significantly reduce anemia in pregnant women and the incidence of low birth weight [7,8]. Protection by treated nets is achieved by the nets acting as a barrier to blood feeding mosquitoes and repelling or killing mosquitoes that are attracted to feed.

However, the effect of conventionally treated nets on malaria vector mosquitoes will diminish as a result of repeated washes, deposit of smoke and abrasion when handled by net owners [9,10]. As a consequence, conventionally treated nets must be retreated every 6–12 months to maintain their efficacy. Recently, the technology for long-lasting insecticide-treated nets (LLINs) has been developed to overcome the problems of re-treatment rates encountered in poor and rural communities. LLINs are treated at factory level by a process that binds or incorporates insecticide into the fibers. Currently LLINs are expected to retain biological activity for at least 20 standard WHO recommended washes and three years of recommended use under field conditions [11].

PermaNet^® ^is an LLINs treated with deltamethrin at a dose of 55 mg a.i./m^2 ^(Vestergaard Frandsen, Denmark) and is recommended by WHO for malaria control. The present study aimed to assess the utilization and retention of nets in Buie and Fentalie districts and monitor the bio-efficacy of nets that have been washed in field conditions by villagers.

## Methods

### Study areas and population

The study was carried out in July, 2008 in six villages of Buie and Fentalie districts. Buie district is within Southern Nations and Nationalities People Region and its administrative capital, Buie, is about 114 Km from Addis Ababa. Three villages, namely *Kella Zuria, Gogitie-2, Gogitie-3*, that are located at the periphery of the town were selected for the study. Population of the study villages were mainly *Guragie *ethnic group engaged in subsistence farming. The majority of houses were made of iron roofs with mud walls while few houses were made of thatch roofs and mud walls. Fentalie district is an area situated in the upper and middle awash valley (700 m a.s.l.) about 190 km eastwards from Addis Ababa. Three villages about 2 km from Metehara town called *Gelcha, Busie *and *Algie *villages were selected for the study. According to the census of 1994, the population of Fentalie district was estimated to be 60,648. Population of the villages were mainly *Oromo *ethnic group engaged in raising cattle and subsistence farming of maize and sorghum. Houses are made of thatch roofs with mud walls.

Both districts are moderately malarious and PermaNet^® ^nets were distributed to malarious villages of Buie and Fentalie districts in addition to one round of indoor residual spraying of DDT by the District Health offices (Personal communication, District Health office). Distribution of PermaNet^® ^was carried out in 2005 and 2006 season by the Districts Health Offices.

### Study design and sampling methods

Selection of the two districts was based on history of distribution of LLINs and variation on housing construction and ethnic background of residents that may affect usage of nets. Villages were selected based on accessibility and proximity to the district capital. In each village, three or more clusters of houses were selected. A sample size of 20 households was considered and every 2^nd ^households were sampled from a reference point of the clusters for interviewing. All households selected had received one or more PermaNet^® ^nets and if a household chosen did not have PermaNet^®^, the next household was selected.

### Data collection, management and analysis

Data were collected in the months of July and August, 2008. Data on the interview were collected by the authors using locally recruited interpreters. Household heads or their representatives were interviewed. The major variables included in the questionnaire were the availability of nets in households, number of nets used in households, number of nets damaged or have holes and utilization of nets by hanging inside houses. Data entered into SPSS 14 software was cleaned by cross checking with responses obtained from the questionnaire. Appropriate analysis was made using SPSS 14 software.

### Bio-efficacy test

Data collection on the bio-efficacy was done using susceptible colony of *An. arabiensis *mosquitoes to deltamethrin according to WHO cone bioassay [12]. The test was carried out using mosquitoes reared in the laboratory by allowing adult female mosquitoes to feed on guinea pigs and eggs collected on moist filter paper were immersed in a bowl of distilled water, until they hatch into larvae. The colony was maintained under laboratory condition by feeding larvae with bakers yeast at a temperature of 26°C.

Two- to three-day old, non blood-fed, female *An. arabiensis *mosquitoes were exposed for three minutes on a used PermaNet^® ^collected from villages according to the standard WHO test procedures [12]. Knock-down results were obtained by counting the number of mosquitoes that were lying down after 60 minutes of exposure. Mortality of mosquitoes was also recorded after a 24-hour holding period. Control mortality was checked by exposing mosquitoes to untreated nets (SAFI NET produced by A to Z manufacturers) of equal replicates were used as controls. When control mortality was between 5–20%, mortality was corrected by Abbott's formula. Tests were repeated when the control mortality was more than 20%. A total of eight used nets collected from each of the villages was used for the bio-efficacy study. The owners of nets were provided with new nets as replacements.

## Results

### Retention of PermaNet^® ^nets

A total of 119 household owners were interviewed during the study while one of the house owner was unavailable. The rate of retention of nets that were distributed in 2005 and 2006 season was 72% (Table 1). Among the respondents, 42% and 46.2% had one or two PermaNet^® ^nets inside their households (Table 2). Since distribution a total of 60.5% the nets had been damaged by having holes as shown in Table 2.

**Table 1:** Status of retention of PermaNet^® ^nets in Buie and Fentalie districts, July 2008

Number of nets	Number of households received	Total number of nets	Nets remaining	Number of households with nets	Total number of nets remaining
1	28	28	1	50	50

2	76	152	2	55	110

3	13	39	3	1	3

4	2	8	4	0	0

*Total*	119	227	Total	119	163

**Table 2:** PermaNet^® ^nets that were observed to have holes among respondents of Buie and Fentalie districts

Status of nets	Number (%)
Without holes	47 (39.5)

1 net damaged	44 (37.0)

2 nets damaged	26 (21.8)

3 nets damaged	2 (1.7)

### Utilization

A total of 64.7% of the interviewees have reported that they sleep under nets every night (Table 3). This was later confirmed by observations of the nets that were hanged inside houses and 50.4% of the households were observed with hanged nets inside houses (Table 3). Regarding knowledge of household heads on the use of nets, 82.4% of respondents claimed that they use the nets for protection from mosquito bites (Table 4). Only 14.3% of the respondents had the knowledge that their nets would protect them from malaria. A total of 62.2% of households also claimed that their children slept under nets during the previous night (Table 4).

**Table 3:** Usage of nets in households and bioassay test results in Buie and Fentalie districts

Frequency of use	Number (%)	Status of net usage	Number (%)
Always	77 (64.7)	Hanged	60 (50.4)

Sometimes	42 (35.3)	Not hanged	44(37.0)
		
		No net	13(10.9)
		
		Unknown	2(1.7)

Bioassay test results	District	Mean KD	Mean mortality
	
	Buie	94%	72.15%
	
	Fentalie	100%	67.0%

**Table 4:** knowledge of respondents on the purpose of using nets and age groups sleeping under nets in Buie and Fentalie districts

Purpose of using nets	Number (%)	Sleeping under net	
		
		Age group	Number (%)
Protect from malaria	17 (14.3)	< 5 year	74 (62.2)

Protect from mosquito bite	98 (82.4)	> 5 year	30 (25.2)

Not known	4 (3.4)	unknown	1 (0.8)

### Bio-efficacy

Table 3 shows results of the bioassay tests. The percentage of mean knock down after 1 hr exposure was 94% and 100%, while mean mortality result after 24 hr holding period was 72.2% and 67% for Buie and Fentalie districts respectively. No knock-down effect was observed for the control replicates.

## Discussion

In malaria endemic countries, the use of insecticide-treated nets is being promoted as an effective method for reducing malaria transmission risk [13]. However the efficacy of treated nets or LLIN, such as PermaNet^® ^nets, for malaria control depends on user acceptability, physical lifespan and bioavailability of insecticide on nets among others [14]. Unlike many African countries, the distribution of nets for free or using social marketing has only been introduced recently in Ethiopia, through WHO, UNICEF, NGOs and Ministry of Health [15]. Utilization of nets from responses of household heads and observation of hanged net inside houses was satisfactory. However, low utilization of nets in urban and peri-urban communities was reported in Ethiopia [15]. Hassan *et al *[16] have reported low utilization of nets by target groups in east Sudan compared to the rate of utilization of nets in nearby countries, such as Eritrea [17]. The study revealed that knowledge of residents' on the purposes of using nets was very high. Although educational status of households was not addressed in the present study, the effect of education on increased use of nets was reported [16].

A moderately high retention of LLIN by owners of nets was observed which is comparable to the report of Hassan *et al *[16] for east Sudan. Although the authors have observed nets that were torn apart and hanged on cattle enclosures, residents in general have a high regard for LLIN as a malaria prevention tool. Some of the residents explained loss and damage of nets they were given saying that the smell of the insecticide and effect on bedbugs and cockroaches had diminished after one year of usage and as a consequence they felt it is not effective for killing mosquitoes. Previous studies also indicated the impact of nets on nuisance insects such as bedbugs and fleas as factors that influence the use of nets by owners. [18].

One of the key parameters to assess effectiveness of nets is to evaluate them under varying eco-cultural as well as epidemiological settings [14]. The bio-efficacy of PermaNet^® ^nets was evaluated using the WHO criteria of 95% knock down and >80% morality rates from the bioassay after 24 hrs [9,10]. Accordingly the observed mean knock down after 1 hr exposure was highly satisfactory. Possible explanations for the observed moderately low bio-efficacy results in this study could be: 1) some of the nets that were evaluated were distributed in 2005, which implies that the nets were in use for more than three years by the time they were collected for the bioassay. 2) decline of deltamethrin available on the surface of the nets due to repeated washing and, deposit of dust and soot because residents of the villages carry out both cooking and sleepig in the same room [19,20]. Considering that the nets are expected to be effective for three years, it is reasonable to conclude that the nets were effective in preventing mosquito bites based on knock-down and exposure mortality results. Similarly, the superior performance of PermaNet^® ^nets is reported in a multi-country study carried out by Graham *et al *[14] and Kroeger *et al *[21]. Kroger *et al *[21] have concluded that PermaNet^® ^nets continued to be effective for at least three years from the bioassay results carried out on *Anopheles nuneztovari *and *Anopheles rangeli *mosquitoes and indicated that 80% mortality could be achieved with 3–5 mg deltamethrin/m^2^.

Though the present study reports a moderately high retention and effectiveness of nets, it should be worth noting that a comprehensive country-wide assessment on the retention rate and efficacy of PermaNet^® ^nets needs to be carried out before making any generalization on the effectiveness of PermaNet^® ^nets.

## Competing interests

The authors declare that they have no competing interests.

## Authors' contributions

MF has participated in the designing of the study, data collection, analysis and drafting of the manuscript. MB participated in the designing of the study, data collection and revision of the manuscript. CG participated in data collection and bio-efficacy of the study. All authors' have approved the final manuscript.
